# Antibacterial potential of biosynthesized silver nanoparticles using *Nepeta sessilifolia* Bunge and *Salvia hydrangea* DC. ex Benth. extracts from the natural habitats of Iran's Rangelands

**DOI:** 10.1186/s12906-023-04101-w

**Published:** 2023-08-24

**Authors:** Mansureh Ghavam

**Affiliations:** Department of Nature Engineering, Faculty of Natural Resources and Earth Sciences, University of Kashan, Kashan, Iran

**Keywords:** Biosynthesis, Extract, Natural antimicrobial, Nanoparticles, Rangelands of Iran

## Abstract

**Background:**

Nowadays, the use of herbal extracts for the production of nanoparticles has attracted a lot of attention due to the fast reaction, economy, and compatibility with the environment. The aim of the present study is the biosynthesis of silver nanoparticles from the extracts of *Nepeta sessilifolia* Bunge and *Salvia hydrangea* DC. ex Benth. and their antibacterial activity was measured.

**Methods:**

For this purpose, the flowering branch of *N. sessilifolia* and the flower of *S. hydrangea* were randomly collected from three places, respectively, from the rangelands of Aqdash Mountain and Biabe in Isfahan province, Iran in May 2021. After extracting aqueous extracts by hot method, silver nanoparticles were synthesized by the biological method. Green synthesized silver nanoparticles were analyzed by UV–Vis spectroscopy, XRD, FTIR, and FESEM-EDAX. The antibacterial effect was evaluated by diffusion method in agar and determination of minimum growth inhibitory and lethal concentration (MIC and MBC) by dilution method in liquid culture medium.

**Results:**

Based on the results of UV–Vis spectroscopy, silver nanoparticles synthesized from *N. sessilifolia* and *S. hydrangea* had distinct absorption peaks at wavelengths of 407 to 424 nm and 414 to 415 nm, respectively. The crystalline nature of these synthetic silver nanoparticles was confirmed by XRD. FESEM analysis showed that the size of biosynthesized silver nanoparticles from *N. sessilifolia* and *S. hydrangea* extracts were 10–50 nm and 10–80 nm, respectively, and were cubic. The results of diffusion in agar showed that the largest diameter of the growth inhibition zone belonging to the synthetic silver nanoparticles from both extracts of *N. sessilifolia* (~ 26.00 mm) and *S. hydrangea* (~ 23.50 mm) was against Gram-positive bacteria *Staphylococcus aureus*. The most vigorous killing activity by synthetic silver nanoparticles from *N. sessilifolia* extract was against *Klebsiella pneumoniae* with a value of 250 μg/mL, two times stronger than rifampin.

**Conclusion:**

Therefore, the studied extracts can be suitable options for fast and safe green synthesis of silver nanoparticles effective against some bacterial strains. These synthetic silver nanoparticles can be used as possible options and have strong potential for the production of natural antibiotics.

## Introduction

Nanotechnology is one of the essential fields in modern science, which deals with synthesizing particles with dimensions in the range of (1–100) nanometers. [1, 2]. Nowadays, metallic nanoparticles (NPs) have received specific attention due to their exceptional physical and chemical, photocatalytic, antibacterial, and magnetic properties [3, 4]. Among all metals, silver nanoparticles have attracted much attention [5, 6]. In recent years, the attention of scientists has been drawn to the antibacterial properties of silver nanoparticles. Silver is a safe inorganic antibacterial agent. Among different silver salts, silver nanoparticles are known as the most suitable candidate for eliminating pathogens [7]. Monovalent silver compounds have been widely used for decades as a treatment for bacterial infections, and studies have shown that silver nanoparticles also have these properties [8]. The toxicity of silver nanoparticles for microorganisms is far more than for human cells. Due to the higher surface-to-volume ratio of nanoparticles compared to bulk metallic silver, the antibacterial properties of silver nanoparticles are much higher [9]. Silver nanoparticles are synthesized by reducing solutions, thermal decomposition of silver compounds, synthesis with the help of microwaves, synthesis with the help of laser, and biological regeneration method [10]. substantial biological resources; Including bacteria, algae, fungi, primary and higher plants, and their products can be used in the synthesis of nanoparticles [11]. The synthesis of nanoparticles from plants has a specific advantage compared to microorganisms, which is the presence of different biomolecules in plants that are responsible for stabilization and act as covering agents for the synthesis of nanoparticles [12, 13]. Various reports have also stated that biosynthesized silver nanoparticles have different biological properties, including antimicrobial [14, 15], antioxidant [16], healing [15], and anticancer [17] properties.

In different parts of the world, native species of the Lamiaceae family, are used by local people in traditional medicine and are usually used as a treatment for gastrointestinal infections [18]. Studies have shown that many plants of the Lamiaceae family have antimicrobial effects [19, 20].

From this family, the genus *Nepeta* includes about 300 species that are widely distributed in Eurasia. 75 species of this genus have been identified in Iran, of which 40 species are native to Iran [21]. 16 species of this genus are used to treat various diseases as culinary or industrial plants [22]. Many species of this genus are used in folk medicine as bacteriostatic, antitussive, antiasthmatic, antispasmodic, antiseptic, and also against skin disorders such as eczema [23, 24]. Some types of native Iranian *Nepeta* are used in Iranian folk and traditional medicine to treat nervous, respiratory, and digestive disorders [25]. *Nepeta sessilifolia* Bunge species (*Lophanthus sessilifolius* (Bunge) Levin) is an exclusive species of Iran, which is distinctly different from other *Nepeta* species with its wingless kaolin leaves. In addition, this species has flowers with long stamens. It grows on the rocky slopes of the mountains in the west and center of Iran, and its flowers appear between June and July [26]. The antimicrobial potency of some *Nepeta* species extracts has been reported [24, 27, 28]. Until now, the synthesis of silver nanoparticles from the extract of some species of this genus such as *Nepeta deflersiana* Schweinf. ex Hedge [29] and *Nepeta leucophylla* Benth. [30] has been registered.

*Salvia* is the largest genus of the Lamiaceae family, with more than 900 species found worldwide. Among these, about 61 native species grow wild in Iran [26], of which 17 species are exclusive to Iran [31]. Since ancient times, *salvia* species have been used to treat more than 60 different diseases, from pain to epilepsy, and mainly to treat colds, bronchitis, tuberculosis, bleeding, and menstrual disorders [32, 33]. In Iranian folk medicine, a wide range of biological activities of crude extracts, essential oils, and purified compounds of *salvia* species have been reported [34]. *Salvia hydrangea* DC. ex Benth. It is a plant with the local name of Gol-e-Aruneh, which is a woody shrub with leaves with comb divisions, and a mace-like inflorescence. Purple to pink calyx, pink to red calyx 22 to 28 mm [35]. Among its most important medicinal uses, we can mention its anti-inflammatory, antispasmodic, antiflatulent, and soothing effects [36]. Its aerial parts are used in Iranian folk medicine as an anti-inflammatory, antispasmodic, and disinfectant, and its flower infusion is used to treat colds [37]. In ethnobotanical surveys in Iran, it has been reported that the people of northeastern Khuzestan province use the decoction of the aerial parts of this plant to treat colds [38]. and the people of the Gardane Rukh region of Chaharmahal and Bakhtiari provinces for anti-flatulence, anti-rheumatism, and colds are used [39]. The antimicrobial activity of *S. hydrangea* extract has been recorded [40]. So far, the synthesis of silver nanoparticles with a diameter of less than 20 nm from S. *hydrangea* extract on the substrate of peach kernel skin has been reported as an effective catalyst [41].

According to the history of the traditional use of these two species in Iran and their reported biological effects, seemingly they are possible natural options with the potential for the synthesis of silver nanoparticles effectively against many bacteria. Therefore, this study was designed and implemented for the first time with the aim of green synthesis of silver nanoparticles and measuring and comparing the antibacterial activity of synthetic silver nanoparticles and extracts of these species (collected from natural habitats of pastures).

## Materials and methods

### Selection of plants and preparation of the aqueous extracts

The flowering branch of *N. sessilifolia* and the flower of S. *hydrangea* from three points randomly from different bases (100 bases in each area) respectively from the Aqdash Mountain (longitude: 43˚29ʹ10ʺ and latitude: 36˚60ʹ955ʺ). and Biabe region (longitude: 46˚48ʹ91ʺ and latitude: 36˚54ʹ087ʺ) of Fereidan City located in Isfahan province, Iran, they were collected in May 2021. Plant materials were washed several times with sterile distilled water to remove impurities and foreign agents. The washed plant materials were spread on flat surfaces in a dark environment at a temperature of 25 °C until they dried. A complete sample of each species was collected and pressed and dried. These species were identified and verified by Mansureh and were recorded and kept under codes 1019 and 1110 in the herbarium of the Faculty of Natural Resources and Earth Sciences of the University of Kashan, Kashan, Iran [42, 43].

The dried plant material was completely powdered by an electric mill. Then 10 g of powder of each species was added to 100 mL of deionized water and heated and stirred for 10 min at 80°C. Then, the extracts were filtered through Whatman No. 1 filter paper and centrifuged at 4000 rpm/min (Herolab/Higen, Germany). The supernatant was stored as a reducing and stabilizing agent for the synthesis of silver nanoparticles at a temperature of 4°C for further use [44].

In another part, crude extracts were concentrated using a rotary evaporator (controlled rotary evaporator with a water bath, model Heidolph Laborota 4003). The concentrated extracts were transferred into a Petri dish and kept in a fan oven at a temperature of 45°C until the complete evaporation of the solvent, and then to a vacuum oven (temperature of 35°C and pressure of 20 mL of mercury) were transferred. After 72 h, the dried extracts were separated from the plates using a spatula. The yield of extract was calculated based on the amount of dry extract obtained in 100 g of the dry plant. Dry extracts were transferred to dark containers with lids and kept at a temperature of 4°C until the next test [45].

### DPPH Radical scavenging activity

In this study, the antioxidant activity of the extract was investigated by measuring the DPPH radical reduction capacity. To prepare the base solution, 25 mg of each extract was weighed and poured into a 25 mL volumetric flask and made up to volume with methanol. In this way, a stock solution with a concentration of 1 mg/mL was prepared for each of the samples. Then, solutions with concentrations of 0.8, 0.5, 0.25, 0.1, 5 × 10^–2^, 5 × 10^–3^, and 5 × 10^–4^, mg/mL of a stock solution were prepared. Then one milliliter of each of the above solutions from dilute to thick was poured into the respective dark volumetric flask. After that, one milliliter of DPPH solution was added to each of the dark balloons and kept at room temperature for half an hour. After 30 min, the prepared solutions were read at a wavelength of 517 nm with a UV/vis spectrophotometer. In the end, after calculating the percentage of inhibition, the logarithm of the concentration was plotted in the Excel program, and the IC50 was calculated in mg/mL from the drawn graph. The inhibition percentage was calculated with the following Eq. 1:

1 $$\mathrm{Percent}\;\mathrm{inhibition}=\frac{sample\;absorption\;rate-control\;absorption\;rate}{sample\;absorption\;rate}$$

### Synthesis of silver nanoparticles

To prepare silver nanoparticles (AgNPs) of each plant, 10 mL of each extract was added dropwise to 90 mL of 1 mM silver nitrate (AgNO3) aqueous solution with continuous stirring. Then, 10 mL of 1.0 M NaOH aqueous solution was slowly added drop by drop to the resulting mixture. The resulting mixture was continuously stirred by a shaker for 30 minutes at room temperature. During the synthesis, with time, due to the surface plasmon resonance (SPR) stimulation, a gradual color change from light yellow to dark brown was observed in the reaction mixture indicating the biological reduction of Ag^+^/Ag^0^ using plant extract and the presence of silver nanoparticles. The supernatant was slowly removed by pipette. The deposited sediment was placed in an oven at 80 °C for 24 h to dry completely. The resulting dry powder was stored in closed penicillin jars at room temperature for further experiments [43, 44].

#### UV–vis spectrum

Bioreduction of Ag^+^ ions to Ag^0^ and the formation of silver nanoparticles by measuring the UV-vis spectrum at regular time intervals (two hours, 48 hours, 4 days, 7 days, and 30 days at ambient temperature) with a visible spectrophotometer (UV-Vis-NIR device) Manufactured by Nano Technology Researchers Company) in the range of 300 to 700 nm and a resolution of 1 nm was investigated and recorded [44].

#### X-ray diffraction (XRD)

The reduced silver nanoparticles were investigated by XRD spectroscopy to confirm the crystallinity and nanostructure of the silver particles [46, 47]. With the establishment of Bragg's law, the average distance between the crystal planes can be obtained using the information in the X-ray diffraction pattern [48]. The average crystallization of particles is calculated using the Debye-Scherrer equation (equation 2). L is the estimated particle size in nanometers, θ is the Bragg angle in radians, λ is the length of the X-ray source (1.5406), the angular width at half of the maximum height, and K is the shape coefficient or Scherer's constant (0.9) [49].

2 $$L=\frac{k\lambda}{\beta\;\mathit{cos}\;\theta}$$

#### Fourier-transform infrared spectroscopy (FTIR)

FTIR analysis was used to identify and characterize the biomolecules of the plant extract in the formation and stabilization of silver nanoparticles. For this purpose, purified silver nanoparticles in powder form were manually ground with potassium bromide to make a pellet. The FTIR spectrum was recorded in the wavelength range of 400-4000 cm^-1^ using Magana 550 model FTIR spectrometer (Nicolet, USA) in diffuse reflection mode [44, 50].

#### Field Emission Scanning Electron Microscopy with Energy Dispersive X-Ray Spectroscopy (FESEM-EDAX)

FESEM analysis was used to observe the surface morphology and size distribution of silver nanoparticles. For this purpose, the sample was covered with a thin layer of platinum, and the SEM image was taken by a TeScan-Mira III FESEM device (made in the Czech Republic) [44]. Energy dispersive X-ray spectroscopy (EDAX) is a chemical analytical technique used in conjunction with scanning electron microscopy (SEM) in material analysis. The elemental composition of nanoparticles was determined using energy-dispersive X-ray spectroscopy [50]. This device has field emission films and works in both high vacuum and low vacuum modes (suitable for non-conductive samples). The resolution of this device is up to 1 nm, and its magnification power is up to 1 million times the voltage of 30 kV. This device is equipped with SE (Secondary Electrons), BSE (Back-Scattered Electrons), LVSTD (Low Vacuum Secondary Electron Tescan Detector), and EDX (Energy-dispersive X-Ray Analysis) detectors and the capability of qualitative analysis (type of elements and formation phases) Giver of substance) and quantity (amount and quantity of elements) of the samples.

### Antibacterial activity measurement

#### Preparation and cultivation of studied strains

The clinical strains studied included two Gram-positive bacteria, Staphylococcus* aureus* and *Staphylococcus epidermidis*, and two Gram-negative bacteria, *Klebsiella pneumonia*, and *Pseudomonas aeruginosa*, which were obtained from Iran Scientific and Technological Research Organization (IROST). These bacteria were cultured in Nutrient Agar (NA) medium and heated in an incubator at 37°C for 24 h.

#### Determining the diameter of growth inhibition zone by diffusion method in (Agar well-diffusion method)

First, plates containing Mueller Hinton agar culture medium were prepared. 100 μL of microbial suspensions with turbidity equal to half McFarland were cultured in uniform conditions on the surface of the culture medium. Extracts and synthetic silver nanoparticles were dissolved in dimethylsulfoxide (DMSO) and reached a concentration of 600 mg/mL. Wells with a diameter of 6 mm and a thickness of 4 mm were created in the culture medium, and 10 μL of silver extract/nanoparticles (with a concentration of 600 mg/mL) were added to each well. The plates were kept at 4°C for 2 h and then heated at 37°C for 24 h in a greenhouse. Antibiotics rifampin (5 µg/well) and gentamicin (10 µg/well) were used as standard drugs for positive control under the same test conditions. The diameter of the growth inhibition zone was measured by an antibiogram ruler (in millimeters). To evaluate the reproducibility and ensure the accuracy of the results, for each extract/silver nanoparticle sample, the test was repeated three times, and the diameter of the growth inhibition halo was reported as the mean ± standard deviation [51].

#### Determining the minimum growth inhibitory concentration (MIC)

To determine the MIC of bacterial and yeast strains, a sterile 96/ well microtiter plate, and broth microdilution method were used according to the guidelines of the Clinical, and Laboratory Standards Institute [52]. At first, various dilutions of each extract/synthetic silver nanoparticle were prepared. In this way, a specific amount of synthetic silver extract/nanoparticles was weighed, and a suitable ratio of culture medium and dimethyl sulfoxide solvent was used to prepare the initial stock. So, the initial concentration of 16000 mg/mL was chosen. Then concentrations of 8000, 4000, 2000, 1000, 500, 250, and 125 mg/mL were prepared from the initial concentration. 200 μL of a solution containing 95 μL of brain heart infusion (BHI) broth, 5 μL of microbial suspension with 0.5 McFarland dilution, and 100 μL of one of the different concentrations of extract/synthetic silver nanoparticles were added to each well of the microplate. The gentamicin and rifampin antibiotics were used to compare their inhibitory power with extract/nanoparticles as a positive control. Then the plates were incubated at 37 °C for 24 h. The first concentration of each synthetic silver extract/nanoparticle in which no growth was observed in the corresponding plate was considered MIC. The test was repeated three times for each sample of synthetic silver extract/nanoparticle, and the average of the lowest concentration of synthetic silver extract/nanoparticles that inhibited the growth of bacteria or yeast was reported MIC.

#### Determining the minimum bactericidal concentration (MBC)

To determine the minimum concentration capable of killing bacteria, the same dilution method was used in the liquid culture medium according to the guidelines of the Clinical and Laboratory Standards Institute (CLSI) similar to the above [52]. After 24 h of heating, 5 μL from each of the microplate wells in which there was no growth were inoculated into nutrient agar medium and incubated at 37°C for 24 h. The first concentration of each extract/synthetic silver nanoparticle sample in which no growth was observed in the corresponding plate was considered MBC.

### Statistical analysis

One-way analysis of variance (ANOVA) was used for the statistical analysis of microbial activities in SPSS 22. The difference between the mean values of the data was evaluated using Duncan's post hoc test at a significance level of 1%. All data were expressed as the mean ± standard deviation.

## Results and discussion

### Extract efficiency

The yield of *N. sessilifolia* and *S. hydrangea* extracts was 8.126 ± 0.006 % and 7.576 ± 0.003 %, respectively. So far, there has been no report on the yield of the extract of these species, and therefore, we are the first report [53] showed that the yield depends on the species, organ type, and plant growth stage. It seems that the accumulation of active ingredients in the flowering branch of *N. sessilifolia* was more than in the flower organ of *S. hydrangea*. The amount of active ingredients in medicinal plants changes in different habitats and regions and the reason for this is the fluctuation of the metabolic activity of plants under the influence of various environmental factors [54]. Differences in habitat characteristics such as height, slope and slope direction, percentage of cover, and other climatic conditions have a great effect on yield [55].

### The antioxidant activity of the extract

The obtained results showed that the IC50 of *N. sessilifolia* and *S. hydrangea* extracts were 154.8817±3.6076 and 50.1187±1.3390 μg/ml, respectively. The amount of antioxidant activity in cases where the IC50 is lower shows better protective effects [56]. Therefore, the antioxidant power of *S. hydrangea* extract was three times stronger than *N. sessilifolia* extract, which is probably due to more phenolic compounds. Different studies show that various species of *Salvia* with high amounts of phenolic compounds have always been studied as one of the most important sources of natural antioxidants [57]. The vital role of phenolic compounds as scavengers of free radicals and the strong correlation between phenolic compounds and antioxidant activity has been confirmed [45, 58]. In previous studies, the IC50 value of *N. sessilifolia* extract from the Kashan region was 105.92 ± 1.39 µg/ml [59] and the IC50 value of *S. hydrangea* extract from the Fars province of Iran was 301.9 ± 43.1 µg/ml [60] has been reported. Antioxidant activity in plant extracts depends on habitat conditions Because these conditions are effective in the synthesis of plant chemicals that have antioxidant properties [61].

### Synthesis of silver nanoparticles

**Fig. 1:**
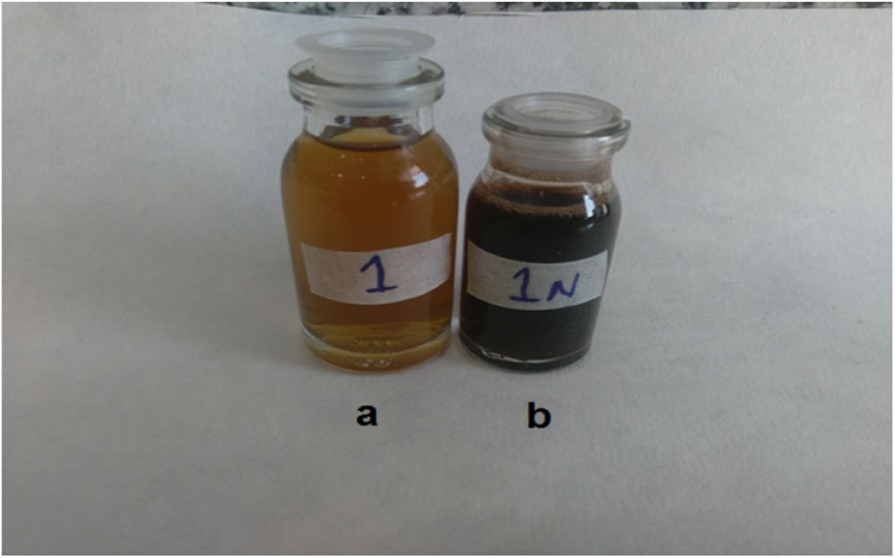
Color change of silver nanoparticle synthesis of *N. sessilifolia* before synthesis b. after synthesis

**Fig. 2:**
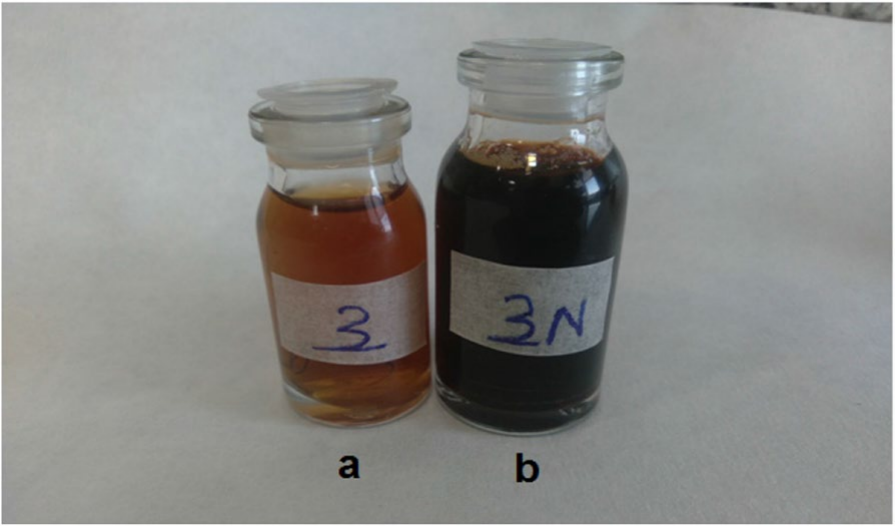
Color change of silver nanoparticle synthesis of *S. hydrangea* before synthesis b. after synthesis

By adding the extracts to the silver nitrate (AgNO3) solution 30 minutes after the start of the reaction, the color of the solution changed from yellow to brown and then dark brown (Figs. 1 and 2). The color change indicates the synthesis of silver nanoparticles and the bioreduction of silver cation (Ag+) to silver (Ag). Similarly, [29] for silver synthesis from *N. deflersiana* extract, [10] for silver synthesis from *N. leucophylla* extract, [41] for silver synthesis from S. *hydrangea* extract, and [62] have reported this color change for silver synthesis from *Salvia miltiorrhiza* Bunge extract. The incidence of brown color could be due to surface plasmons [63]. Silver nitrate is decreased to silver nanoparticles due to the presence of reducing polyphenols and flavonoids in the extracts [64].

### Specifications of silver nanoparticles

#### UV–vis spectroscopy

**Fig. 3:**
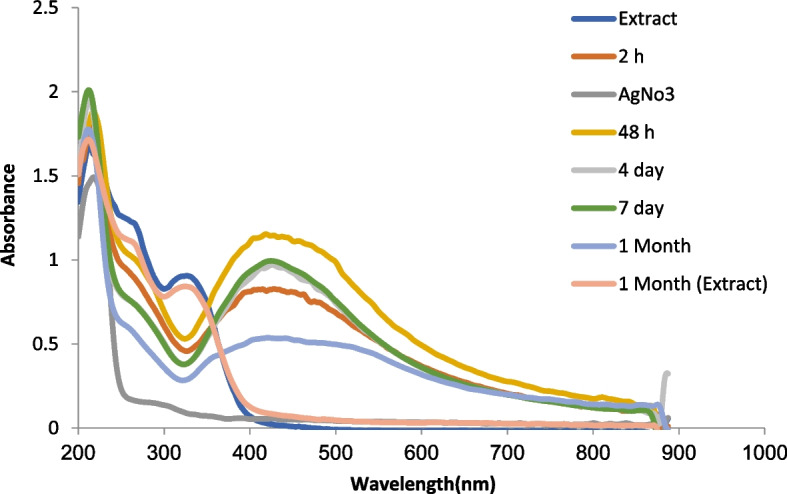
UV–Vis absorption spectrum of silver nanoparticles synthesized from *N. sessilifolia* extract at different times

**Fig. 4:**
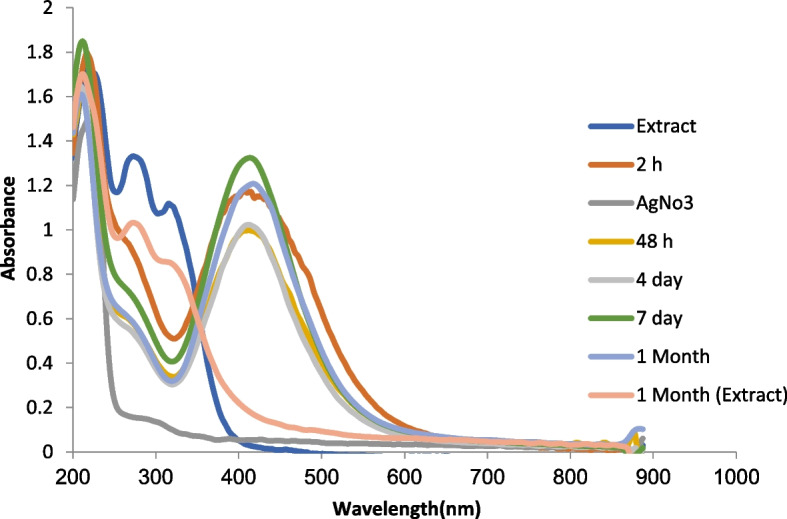
UV–Vis absorption spectrum of silver nanoparticles synthesized from *S. hydrangea* extract at different times

After observing the color change, the production of nanoparticles was confirmed by UV-Vis spectroscopy. The maximum absorption peak in the curves obtained after 2 h, 48 h, 4 d, 7 d, and one month of reaction time, for the solution containing biosynthesized silver nanoparticles with *N. sessilifolia* extract, respectively, at wavelengths of 407 and 418, 424, 424, 420 nm were observed (Fig. 3). The results showed that for the solution containing nanoparticles biosynthesized with *S. hydrangea* extract, the maximum absorption peak in the above time intervals was at the wavelength of 414, 414, 414, 415, and 415 nm, respectively (Fig. 4). The maximum absorption peak in the range of 400 to 450 nm indicates the synthesis of silver nanoparticles. It is related to the surface plasmon resonance of silver nanoparticles, which is related to the induction of free electrons in the nanoparticles [65].

Similarly, the maximum absorption peak of the solution containing synthetic silver nanoparticles with the extract of *Nepeta deflersiana* Schweinf. ex Hedge at a wavelength of 400 [29] and with the extract of *Salvia rhytidea* Benth. It has been reported at a wavelength of 415-400 nm [66]. Based on the results over time, the absorption intensity gradually increased and after 4 days it reached the maximum value, which is in line with the results of [62] for the synthesis of silver from *S. miltiorrhiza* extract. On the other hand, the extract of *N. sessilifolia* and *S. hydrangea* had no peak, which indicates that the extract did not interfere with the spectrum of silver nanoparticles. Similarly, [67] for the extract. *Salsola vermiculata* L. reported no absorption peak in the range of 400 to 500 nm. Probably, the presence of phenolic compounds of *N. sessilifolia* and *S. hydrangea* extracts had an important role in regenerating metal ions and converting them into metal atoms in nanometric dimensions, and stabilizing the synthesized nanoparticles.

#### X-ray diffraction (XRD)

**Fig. 5:**
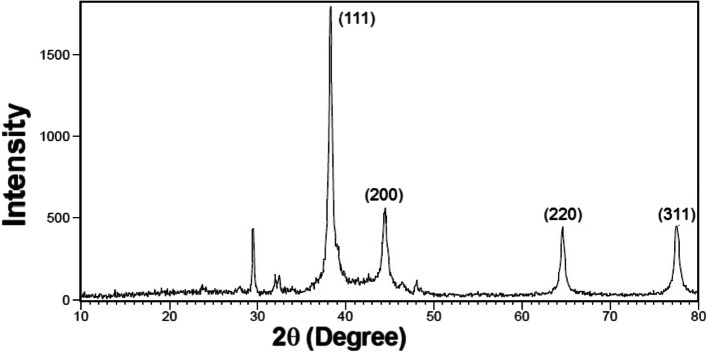
XRD spectrum of silver nanoparticles synthesized from *N. sessilifolia* extract

**Fig. 6:**
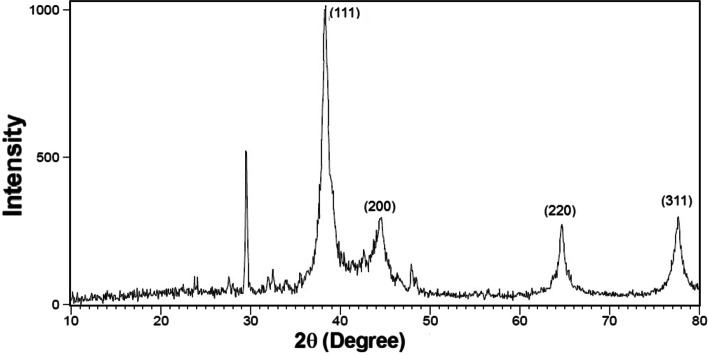
XRD spectrum of silver nanoparticles synthesized from *S. hydrangea* extract

Figures 5 and 6 show the X-ray diffraction patterns obtained from silver nanoparticles using *N. sessilifolia* and *S. hydrangea* extracts. Sharp peaks at 2θ angles equal to 38.2807 ˚, 44.4693 ˚, 64.6179 ˚, and 77.5470 ˚ in the XRD pattern of silver particles synthesized from *N. sessilifolia* extract and 38.3110 ˚, 44.5268 ˚, 64.6679 ˚ and 77.6537 ˚ in the XRD pattern of silver particles synthesized from *S. sessilifolia* extract *S. hydrangea* are responsible for crystallographic Bragg reflection planes (111), (200), (220), and (311). This confirmed the crystalline nature of silver nanoparticles [68] and is in good agreement with the results of the Joint Committee on Powder Diffraction Standards (JCPDS) [69]. A similar result for the XRD pattern was obtained from synthetic silver nanoparticles from *S. rhytidea* extract. [66] and *N. leucophylla* [30] have been registered. The non-indicative peaks at 23˚, 27˚ and 29˚, 32˚ and 35˚, and 47˚and 48˚ in the XRD pattern of synthetic silver nanoparticles can indicate the formation of crystals of organic molecules [69], while the corresponding peak to impurities or other compounds in the XRD pattern obtained from silver nanoparticles. *S. hydrangea* [41] was not reported, which contradicts our results. The small width of the diffraction pattern at half maximum peak (111) indicates the large crystal size of silver nanoparticles [70]. The size of silver particles synthesized from *N. sessilifolia* and *S. hydrangea* extract was equal to 4.1389 and 3.0949 nm, respectively, according to the Debye-Scherrer equation, which is comparable to the size determined from the transmission electron micrograph. The average particle size of synthetic silver nanoparticles from N. leucophylla extract is 3.25 to 17.26 nm [30], from *N. deflersiana* extract 33 nm [29]. from and *S. rhytidea* extract. 24.82-19.65 nm [66] was reported, which is not consistent with the present results.

#### FTIR analysis

**Fig. 7:**
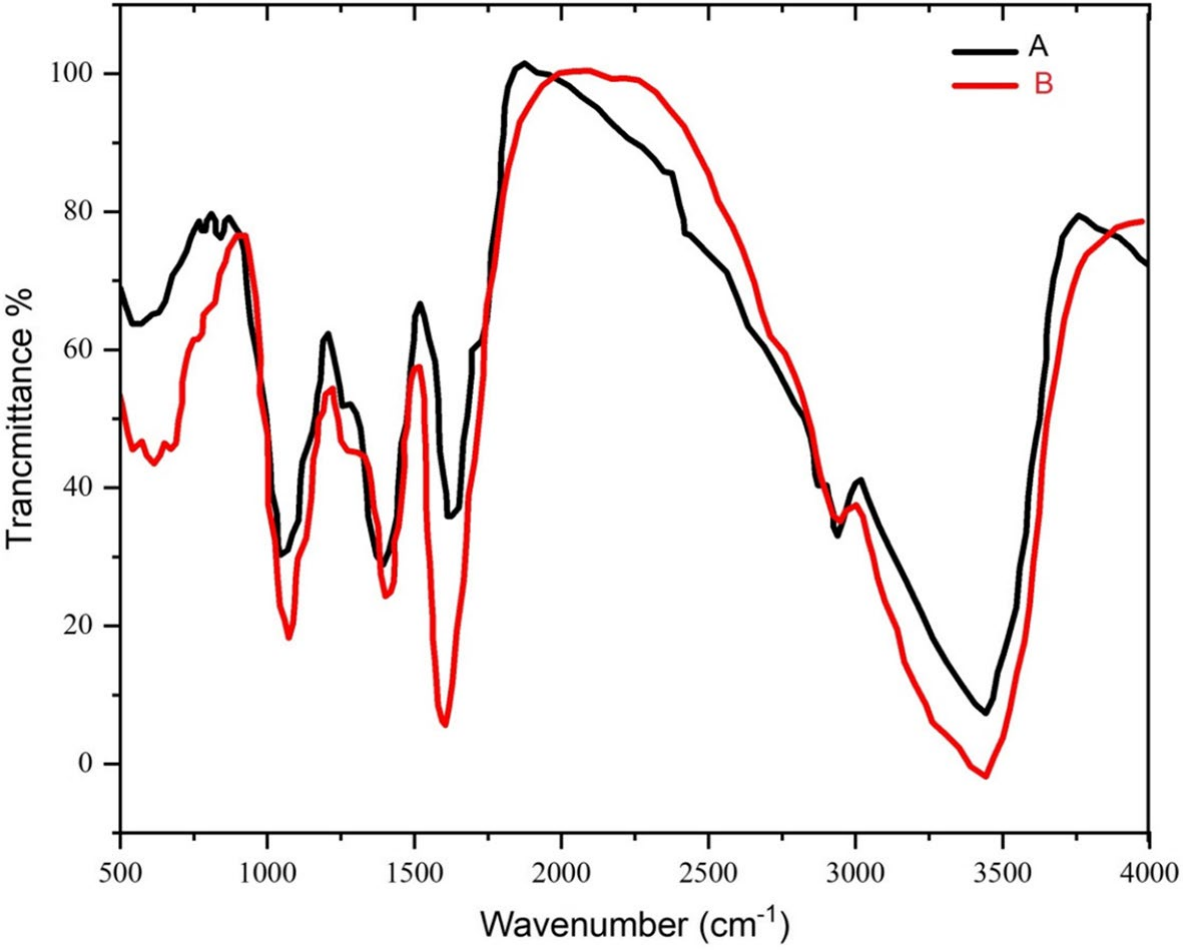
FTIR spectrum of silver nanoparticles synthesized and *N. sessilifolia* extract. A: FTIR spectrum of *N. sessilifolia* extract, B: FTIR spectrum of synthetic silver nanoparticles

**Fig. 8:**
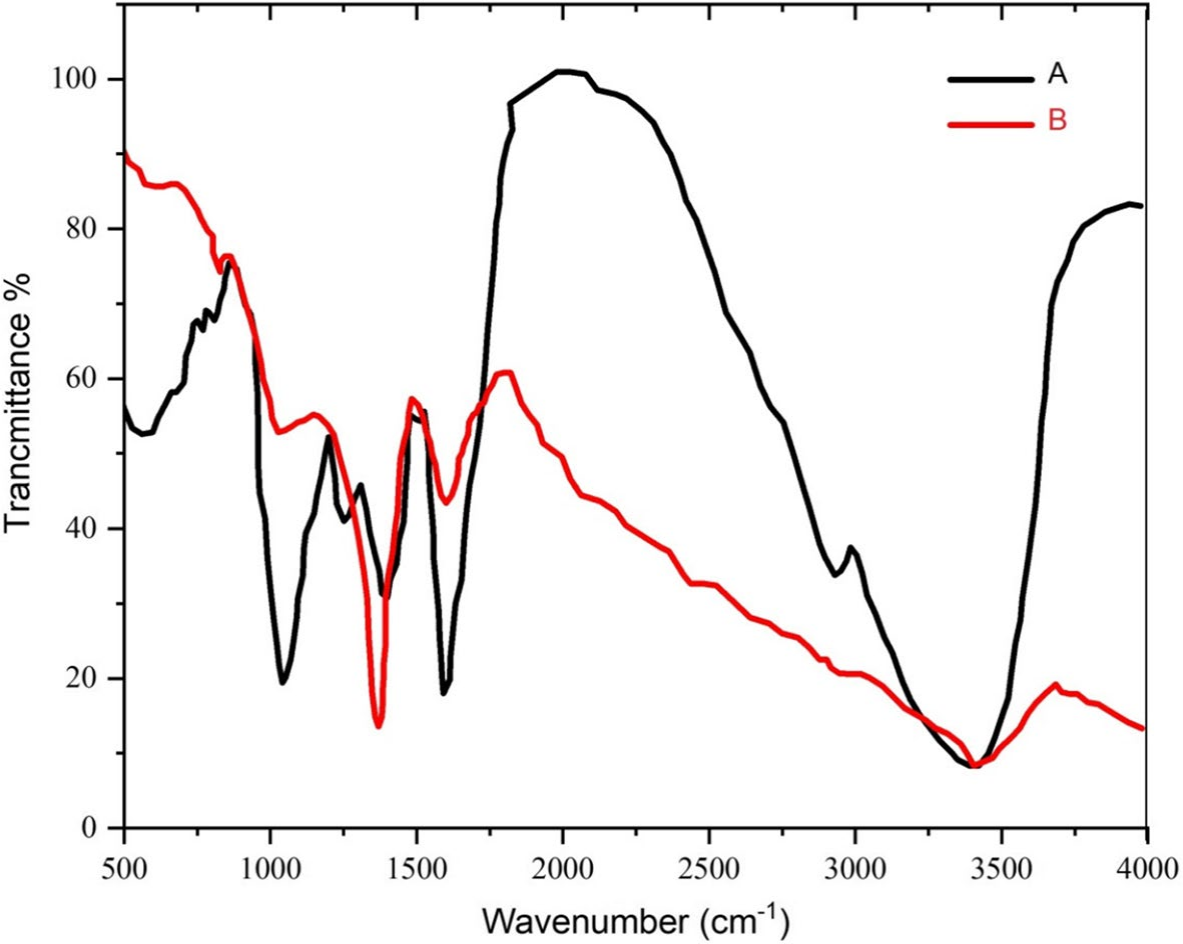
FTIR spectrum of silver nanoparticles synthesized and *S. hydrangea* extract. A: FTIR spectrum of *S. hydrangea* extract, B: FTIR spectrum of synthetic silver nanoparticles

Various functional groups responsible for the reduction and stabilization of biosynthesized nanoparticles were determined using the FTIR technique [71]. The retention spectrum was recorded between 400 cm^-1^ and 4000 cm^-1^ wavelength (Figs. 7 and 8). The FTIR spectrum of *N. sessilifolia* extract showed 9 absorption peaks in the scope 3401.97 cm^-1^ to 553.08 cm^-1^ (Fig. 7-A). Meanwhile, 10 absorption peaks in the wavelength range of 3389.06 cm^-1^ to 613.44 cm^-1^ were reported in the FTIR spectrum of *S. hydrangea* extract (Fig. 8-A). The broad absorption peaks at 3401.97 cm^-1^ and 3389.06 cm^-1^ were assigned to the O-H stretching of the phenolic compounds of the extracts [72]. Therefore, it can be said that the biosynthesized silver nanoparticles were stabilized by the polyphenol compounds of the extracts, which confirms the antioxidant activity of the extracts. Previous studies showed that the phenolic functional groups of the extract of *Salvia officinalis* L. [73], *N. deflersiana* [29], *N. leucophylla* [30], and *S. hydrangea* [41] reduced AgNO3 and stabilized biogenic nanoparticles. The presence of peaks at 2930.73 cm^-1^ and 2930.32 cm^-1^ respectively, in the FTIR spectra of *N. sessilifolia* and *S. hydrangea* extracts confirmed the C-H stretching of alkane, aldehyde, and aromatic alkene, which is consistent with the results of Khodadadi, (2018), *S. hydrangea* extract corresponds to the bands at 1602.10 cm^-1^ and 1606.40 cm^-1^ in the spectrum of *N. sessilifolia* and *S. hydrangea* extracts corresponding to carbonyl stretching (C=O) of amide-I bond and -N-H stretching vibrations. The amide-II bond was the protein [30]. In the spectra of *N. sessilifolia* and *S. hydrangea* extracts, absorption peaks of 1404.75 cm^-1^ and 1404.97 cm^-1^ were assigned to the N-H stretching vibration present in the amide bonds [74]. Weak peaks of 1296.67 and 1270.92 cm^-1^ in the spectra of *N. sessilifolia* and *S. hydrangea* extracts showed C=C bending, which is in line with the spectrum results of *Salvia officinalis* L. extract [73]. The intense peaks of 1078.17 cm^-1^ in the spectrum of *N. sessilifolia* extract and 1058.18 cm^-1^ in the spectrum of *S. hydrangea* extract were related to C-O stretching vibrations, which following the findings of the results of [46] for silver synthesis from the extract of *Thymus trautvetteri* Klokov & Des-Shost. The weak band detected at 820.57 cm^-1^ in the spectrum of *S. hydrangea* extract indicated the C-H bending of alkenes [73]. Weak peaks at 773.05 cm^-1^, 615.32 cm^-1^, and 553.08 cm^-1^ in the spectrum of *N. sessilifolia* extract and 773.67 cm^-1^ and 613.44 cm^-1^ in the spectrum of *S. hydrangea* extract belong to the group Functionality of metal-oxygen bonds was assigned [75]. The FTIR spectrum of synthetic silver nanoparticles from both studied extracts had a consistent process with the spectrum of the extracts (Figs. 7 and 8-B). All the peak changes support the use of the functional groups of the extracts as reducing and stabilizing agents in the biosynthesis of silver nanoparticles [76]. This similarity shows that some remaining parts of plant molecules from the extract remain on the surface of synthesized silver nanoparticles and act as a stabilizing agent. The carbonyl groups of the extract form amino acid residues that lead to the formation of proteins. These proteins have a high strength to bind to metal, thereby acting as a capping agent and preventing their accumulation [77]. Therefore, the reduction of Ag ions can be due to the presence of flavonoids, alcoholic and phenolic compounds, tannins, terpenoids, and glycosides in the extracts [78].

#### FESEM-EDX analysis

##### SEM

**Fig. 9:**
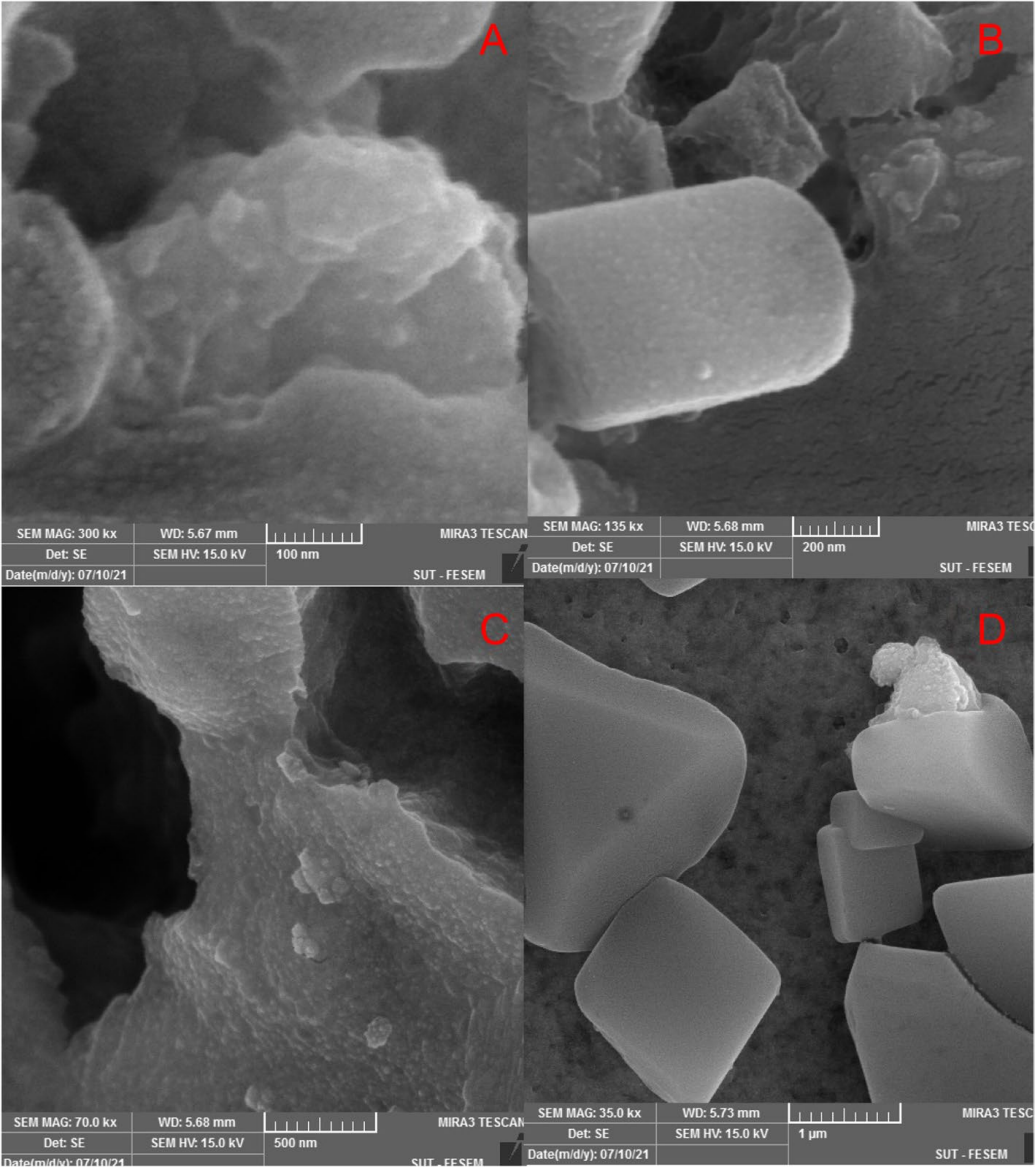
SEM images of nanoparticles synthesized from *N. sessilifolia* extract with different magnifications: **A**- 100 nm, **B**- 200 nm, **C**- 500 nm, **D**- 1 µm

**Fig. 10:**
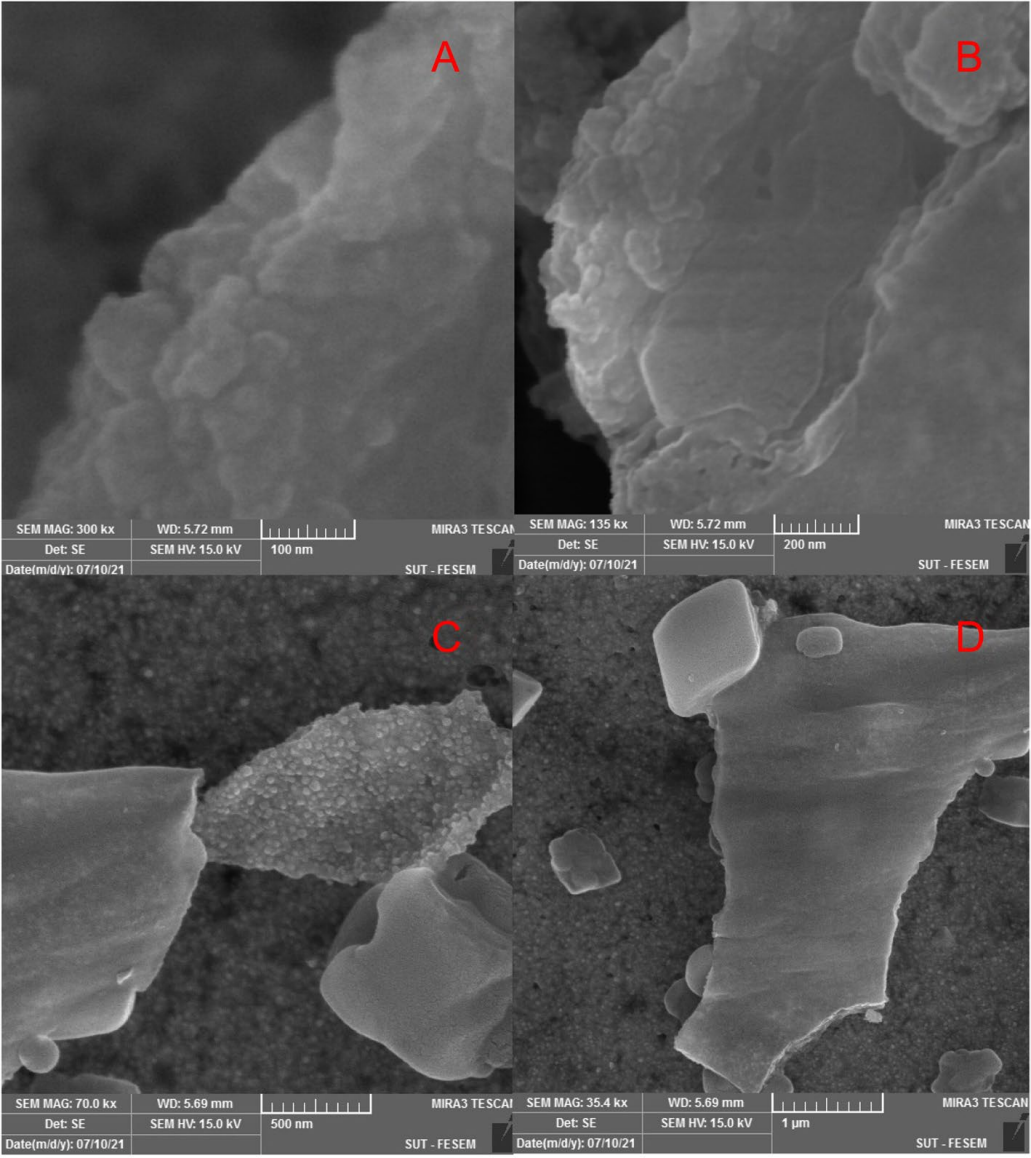
SEM images of nanoparticles synthesized from *S. hydrangea* extract with different magnifications: **A**- 100 nm, **B**- 200 nm, **C**- 500 nm, **D**- 1 µm

FESEM images showed that silver nanoparticles synthesized from both extracts were cubic products. In some areas, the nanoparticles are stacked together [79] (Figs. 9 and 10). In previous studies, the shape of nanoparticles synthesized from *N. deflersiana* [29] and S. hydrangea [41] was recorded as spherical, which is contrary to our results. Some of the larger particles observed may be due to the aggregation of nanoparticles due to solvent evaporation during sample preparation [80]. The average size of synthetic silver particles from *N. sessilifolia* and *S. hydrangea* extracts was 10–50 nm and 10–80 nm, respectively. Similarly, the average size of synthetic silver nanoparticles from *S. rhytidea* extract is about 20–25 nm [66], *S. Officinalis* 17.6 nm [73], *N. deflersiana* 33 nm [29], and *S. hydrangea* 20 nm (Khodadadi, 2018) were reported, which is in line with the present results.

##### EDAX

**Fig. 11:**
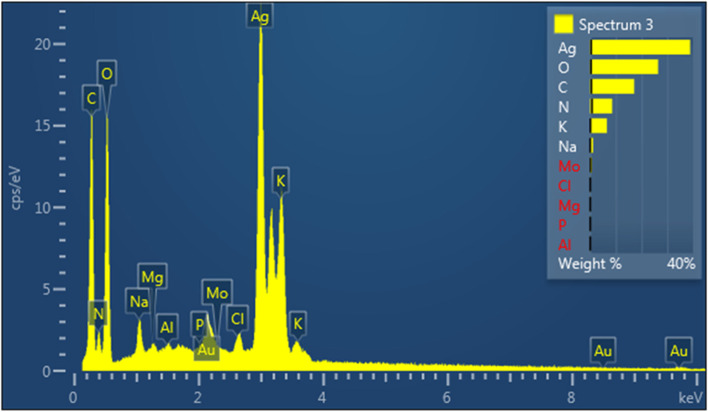
SEM–EDX image of nanoparticles synthesized from *N. sessilifolia* extract

**Fig. 12:**
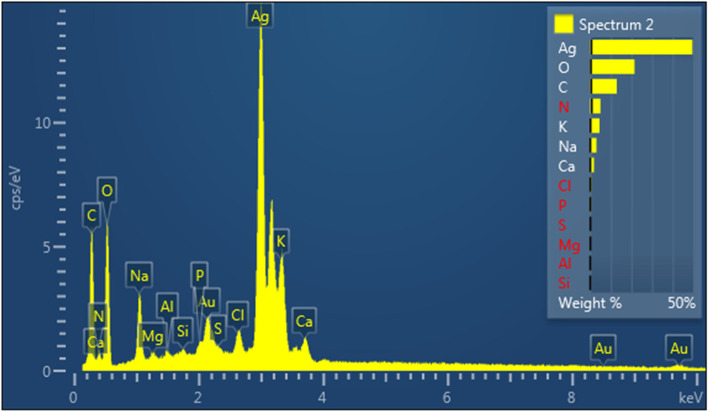
SEM–EDX image of nanoparticles synthesized from *S. hydrangea* extract

Energy dispersive X-ray spectroscopy (EDAX) was used to detect the presence of elemental silver. The EDAX spectrum shows the essential signal in the silver region, which indicates the formation of silver nanoparticles from *N. sessilifolia* and *S. hydrangea* extracts (Figs. 11 and 12). The results clearly show an intense signal at around 2.98 keV related to the presence of metallic silver nanocrystals, which occurs due to surface plasmon resonance (SPR) [81]. The weight percentage of a silver element obtained from the extracts of *N. sessilifolia* and *S. hydrangea* was 38.83 wt% and 49.60 wt%, respectively. Similarly, the presence of silver nanoparticles from the extracts of *S. Officinalis* (16.83 wt%) and *N. deflersiana* (93.10 wt%) has been confirmed at around 2.98 keV in the EDAX spectrum [29, 73]. The intense signals at 0.0–0.5 keV were related to O (26.38 and 21.46 wt%) and C (17.14 and 12.89 wt%), which can be caused by plant compounds and the surface of biosynthesized silver nanoparticles [76]. The oxygen peak can be attributed to X-ray emission from free amino groups [82].

### Antibacterial activity

Based on the results of the analysis of variance, there was a significant difference between the growth inhibition zone diameter of synthesized silver nanoparticles, extracts, and control antibiotics on the studied bacteria (*P* ≤ 0.01) (Table 1). The largest diameter of the growth inhibition zone belonging to the synthetic silver nanoparticles from both extracts of *N. sessilifolia* (26.00 ± 0.00 mm and *S. hydrangea* (23.50 ± 0.50 mm)) was against Gram-positive bacteria *S. aureus*, which compared to antibiotics, rifampin (36 ~ mm) and, gentamicin (31 ~ mm) have strong antibacterial activity. Similarly [66], for synthetic silver nanoparticles from *Fumaria parviflora* Lam. reported a growth inhibition zone diameter of 34 ± 0.26 mm against this bacterium. The reason for the greater sensitivity of gram-positive bacteria may be that these bacteria have a single layer in their cell wall, while in Gram-negative bacteria, this wall consists of several layers. In other words, gram-negative bacteria have an outer membrane and a periplasmic space, none of which exist in gram-positive bacteria. The outer membrane of Gram-negative bacteria is known as a barrier for the penetration of many antibiotic molecules. On the other hand, this membrane prevents the penetration of hydrophilic into the bacteria. Periplasmic space also contains many enzymes that can break down foreign molecules that enter outer space [83]. Silver nanoparticles provide good antibacterial properties due to their large surface area relative to volume, which allows for favorable contact with bacterial cells [84]. According to the findings of some researchers, silver nanoparticles with an average size of less than 10 nm have the greatest antimicrobial effect if they are not attached [85], which is per the average size of our synthetic silver nanoparticles. This was while both the studied extracts acted against this bacterium with a growth inhibition zone diameter of 0.00 ± 8.00 mm, significantly weaker than the control antibiotics and synthetic silver nanoparticles. Similar results were obtained by [66] for *S. rhytidea* extract (~ 7mm) and silver nanoparticles synthesized from *S. rhytidea* (~ 20mm) against this bacterium. Perhaps the reason for this can be seen as the fact that silver nanoparticles destabilize the plasma membrane potential, which results in a decrease in the level of ATP inside the cell. This action is performed by targeting the bacterial cell membrane and causes the death of the bacteria [86]. Silver nanoparticles cause the disintegration of the hindering components present in the bacterial outer membrane, which causes the progressive release of molecules such as lipopolysaccharide and purines from the cytoplasmic membrane. Also, silver nanoparticles do not only stick to the surface of the cell membrane but also penetrate the cells. After penetrating the bacterial cell, the silver nanoparticle deactivates its enzymes and causes the death of the bacteria by producing hydrogen peroxide. After sticking to the surface of the cell membrane, silver nanoparticles destroy the respiratory system in the form of enzyme interaction with the bacterial respiratory chain with Ag + [70]. Therefore, it can be concluded that silver nanoparticles have better inhibitory effects against bacteria than extracts.

**Table 1:** Growth inhibition zone diameter of synthesized nanoparticles and extract of *N. sessilifolia*, *S. hydrangea* and antibiotics against microbial strains

strains	DIZ (mm)					
	AgNPs		Extract		Antibiotics	
	*N. sessilifolia*	*S. hydrangea*	*N. sessilifolia*	*S. hydrangea*	Rifampin	Gentamicin
Gram-negative bacteria	14.00 ± 0.00^b^	9.50 ± 0.50^a^	8.00 ± 0.00^d^	8.00 ± 0.00^d^	12.00 ± 0.00^c^	24.00 ± 0.00^a^
*K. pneumoniae*						
*P. aeruginosa*	26.00 ± 0.00^a^	21.00 ± 1.00^c^	ND	ND	11.00 ± 0.00^d^	23.00 ± 0.00^b^
Gram-positive bacteria	27.00 ± 1.00^c^	23.50 ± 0.50^d^	8.00 ± 0.00^e^	8.00 ± 0.00^e^	36.00 ± 0.00^a^	31.00 ± 0.00^b^
*S. aureus*						
*S. epidermidis*	25.00 ± 1.00^c^	22.00 ± 0.00^d^	9.00 ± 0.00^e^	8.50 ± 0.50^f^	46.00 ± 0.00^a^	32.00 ± 0.00^b^

*ND* not determined. Values with different letters are statistically different (Duncan, *p* = 0.01)

Among other significant inhibitory activities of silver nanoparticles synthesized from the extracts of *N. sessilifolia* (26.00 ± 0.00 mm) and *S. hydrangea* (21.00 ± 1.00 mm) was against Gram-negative bacteria *P. aeruginosa*, which was almost twice as strong as rifampin. (~ 11 mm) and were equal to gentamicin (~ 23 mm). Similarly, the diameter of the inhibition zone of silver nanoparticles synthesized from *S. officinalis* has been reported to be 14.27 mm [73]. The presence of a layer of lipopolysaccharide on the outer surface of Gram-negative bacteria, which is rich in negative charges, facilitates the interaction between positively charged silver nanoparticles and these bacterial cells. The attachment of nanoparticles to the cell surface first pierces the wall, and then by entering the nanoparticle into the bacterial cell and causing interference in various metabolic and reproductive pathways, it ultimately leads to the inhibition of the bacteria [87, 88]. On the other hand, the absence of growth inhibition zone diameter by both studied extracts against this bacterium proved the lack of inhibitory ability of these extracts. It has been reported against this bacterium that the diameter of the growth inhibition zone is not created by the extract of some *Nepeta* species such as *Nepeta trachonitica* Post [24] and *Nepeta cataria* L. [89]. Based on the results, the diameter of the inhibition zone of the growth of synthetic silver nanoparticles from *N. sessilifolia* and *S. hydrangea* extracts against Gram-positive bacteria *S. epidermidis* was 25.00 ± 1.00 and 22.00 ± 0.00 mm, respectively, which compared to rifampin (~ 46 mm) and gentamicin (~ 32 mm) have worked relatively strongly. This is while the inhibitory activity of extracts of *N. sessilifolia* (0.00 ± 9.00 mm) and *S. hydrangea*. (0.00 ± 8.00 mm) against this bacterium was many times weaker than the control antibiotics and synthetic silver nanoparticles. As far as we know, there is no report on the inhibitory activity of synthetic silver nanoparticles and extracts against this bacterium. The present study is the first report of the high inhibitory power of synthetic silver nanoparticles against *S. epidermidis* to introduce a significant and promising potential and a possible natural alternative against this bacterium.

Another significant inhibitory activity was related to synthetic silver nanoparticles from *N. sessilifolia* extract with an inhibition zone diameter of 14.00 ± 0.00 against Gram-negative bacteria *K. pneumoniae*, compared to synthetic silver nanoparticles from *S. hydrangea* extract. (Diameter of growth inhibition zone = 9.50 ± 0.50 mm) and the antibiotic rifampin (~ 12 mm) worked significantly stronger [73] recorded the strong inhibitory activity of *S. officinalis* silver nanoparticles (25.18 ± 0.27 mm) against this bacterium, which is contrary to the present results. The results of many kinds of research, based on the possible reactions between nanoparticles and macromolecules of living organisms, show that the difference between the negative charge of the microorganism and the positive charge of the metal nanoparticles acts as an absorbing electromagnet between the microbe and the nanoparticles and causes the nanoparticles to be attached to the cell surface and as a result, It can cause cell death [66]. Finally, a large number of these contacts lead to the oxidation of the surface molecules of microbes and their rapid death [90].

On the other hand, there was no significant difference between the inhibitory activity of the extracts (8.00 ± 0.00 mm); But they had significantly less inhibitory power than rifampin (~ 12 mm) and gentamicin (~ 24 mm). Similarly, the diameter of the growth inhibition zone of *Nepeta laevigata* (D.Don) Hand.-Mazz. 8 mm has been reported [91]. Reducing the particle size of this metal increases its activity and antibacterial effect. The antibacterial effect of silver is due to the slow and continuous release of silver ions. The ratio of the surface to the size of very large silver particles makes the ions easily emitted and destroy more microbes very quickly and effectively. Also, the presence of silver causes loosening and instability of the cell wall and membrane, which makes this cell membrane unstable. Microbial death Another good feature of nanosilver is that the antimicrobial effect of silver does not decrease over time [14]. On the other hand, the findings of the minimum concentration of inhibition and lethality by the dilution method in the liquid culture medium indicated that the MIC and MBC values of *N. sessilifolia* and *S. hydrangea* extracts against all studied bacteria ranged from 8000 to > 16,000 µg/ml. were that they had performed very poorly compared to the control antibiotics (Tables 2 and 3). The lowest MIC value of synthetic silver nanoparticles from N*. sessilifolia* extract against this *P. aeruginosa* bacterium was 125 μg/mL, which is twice that of rifampin (MIC = 31.25 μg/mL) and four times that of gentamicin (MIC = 7.8 μg/mL has performed weaker. Meanwhile, the MBC value of *N. sessilifolia* silver nanoparticles against this bacterium was 1000 μg/mL, which is very weak compared to rifampin (MBC = 15.63 μg/mL) and gentamicin (MBC = 250 μg/mL). Based on previous studies, the MIC and MBC values against this bacterium by silver nanoparticles synthesized from *Allium paradoxum* (M.Bieb.) G.Don are 1.8 and 7.5 μg/mL [92] and by nanoparticles, Synthetic silver from *Origanum majorana* L. 5 and 10 μg/mL [70] has been recorded, which is contrary to the present results. The strongest killing activity by synthetic silver nanoparticles from *N. sessilifolia* extract was against *K. pneumonia* with a value of 250 μg/mL, which was twice as strong as rifampin (MBC = 500 μg/mL). Meanwhile, synthetic silver nanoparticles from *S. hydrangea* extract with an MBC value equal to 1000 μg/mL acted twice weaker than rifampin. Similarly, the MBC value of silver nanoparticles synthesized from the extract of *Scrophularia striata* Boiss. equal to 250 μg/mL have been reported [93], per the present results. The antibacterial properties of silver nanoparticles in gram-negative bacteria depend on the concentration of the nanoparticles and accumulation in the form of pits in the cell wall. These nanoparticles accumulated in the membrane cause membrane permeability and gradual cell death [94].

**Table 2:** Minimum inhibitory concentration (MIC) of synthesized nanoparticles and T. extract and extract of *N. sessilifolia*, *S. hydrangea* and antibiotics against microbial strains

strains	MIC (μg/mL)					
	AgNPs		Extract		Antibiotics	
	*N. sessilifolia*	*S. hydrangea*	*N. sessilifolia*	*S. hydrangea*	Rifampin	Gentamicin
Gram-negative bacteria	250	1000	> 16,000	8000	31.25	7.8
*K. pneumoniae*						
*P. aeruginosa*	125	500	> 16,000	> 16,000	31.25	7.8
Gram-positive bacteria	250	1000	> 16,000	> 16,000	3.9	7.8
*S. aureus*						
*S. epidermidis*	250	1000	> 16,000	> 16,000	1.95	3.9

**Table 3:** Minimum bactericidal concentration (MBC) of synthesized nanoparticles and extract and extract of *N. sessilifolia*, *S. hydrangea* and antibiotics against microbial strains

strains	MBC (μg/mL)					
	AgNPs		Extract		Antibiotics	
	*N. sessilifolia*	*S. hydrangea*	*N. sessilifolia*	*S. hydrangea*	Rifampin	Gentamicin
Gram-negative bacteria	250	1000	> 16,000	> 16,000	500	7.8
*K. pneumoniae*						
*P. aeruginosa*	1000	2000	> 16,000	> 16,000	250	15.63
Gram-positive bacteria	500	1000	> 16,000	> 16,000	3.9	7.8
*S. aureus*						
*S. epidermidis*	250	1000	> 16,000	> 16,000	7.8	3.9

## Conclusion

The present study reports the production of silver nanoparticles from a simple, fast, and eco-friendly route using *N. sessilifolia* and *S. hydrangea* extracts. The studied extracts reduced the aqueous solution of silver nitrate and stabilized the nanoparticles formed in the reaction. Biosynthetic silver nanoparticles were crystalline in nature, cubic in shape, and 10 to 80 nm in size. These synthetic silver nanoparticles have the potential to inhibit the growth of *S. aureus* and *P. aeruginosa*, *S. epidermidis*, and *K. pneumoniae* and were stronger than extracts and some control antibiotics. Two times stronger killing activity than rifampin against *K. pneumoniae* was observed by synthetic silver nanoparticles from *N. sessilifolia* extract, which is a promising and unique potential against this bacterium. Silver nanoparticles produced by the green method have the potential to be used in industries related to human health, such as healthcare, due to the absence of the use of dangerous chemicals. It seems that silver nanoparticles synthesized from these extracts can be a possible option for the treatment or prevention of some infectious diseases of bacterial origin. However, further and additional research should be done in the future to be approved for clinical applications.

## Data Availability

The datasets used and/or analysed during the current study available from the corresponding author on reasonable request.

## Abbreviations

GC/MS: Gas chromatography–mass spectrometry
Μg: Microgram
mL: Miligram
MBC: Minimum bactericide concentration
MIC: Minimal inhibitory concentration
IROST: Iranian Research Organization on Science and Technology
ANOVA: One-way analysis of variance
RI: Retention indices
XRD: X-ray diffraction
FTIR: Fourier-transform infrared spectroscopy
FESEM-EDAX: Field Emission Scanning Electron Microscopy with Energy Dispersive X-Ray Spectroscopy
